# TGF-β1 contributes to CD8^+^ Treg induction through p38 MAPK signaling in ovarian cancer microenvironment

**DOI:** 10.18632/oncotarget.10003

**Published:** 2016-06-14

**Authors:** Meng Wu, Xian Chen, Jianfang Lou, Shuping Zhang, Xiaojie Zhang, Lei Huang, Ruihong Sun, Peijun Huang, Fang Wang, Shiyang Pan

**Affiliations:** ^1^ Department of Laboratory Medicine, The First Affiliated Hospital of Nanjing Medical University, 210029, Nanjing, China; ^2^ National Key Clinical Department of Laboratory Medicine, 210029, Nanjing, China; ^3^ Department of Laboratory Medicine, The Affiliated Children Hospital, Nanjing Medical University, 210029, Nanjing, China

**Keywords:** TGF-β1, p38 MAPK, CD8^+^ Treg, ovarian cancer

## Abstract

CD8^+^ regulatory T cells (Tregs) contribute to cancer progression and immune evasion. We previously reported that CD8^+^ Tregs could be induced *in vitro* by co-culture of CD8^+^ T cells with the OC cell lines SKOV3/A2780. Here, we described the role of TGF-β1 in CD8^+^ Treg induction by the OC microenvironment. OC patients expressed high levels of TGF-β1, as did the co-culture supernatant from CD8^+^ T cells and SKOV3. Additionally, TGF-β1 levels were positively correlated with CD8^+^ Treg percentages in OC. Neutralization experiments, cytokine studies and proliferation assays revealed that the *in vitro*-induced CD8^+^Tregs depended at least partially on up-regulated expression of TGF-β1 to exert their suppressive function. CD8^+^ T cells cultured with SKOV3 exhibited marked activation of p38 MAPK than CD8^+^ T cells cultured alone, which could be inhibited by TGF-β1-neutralizing antibody. Moreover, the p38 specific inhibitor SB203580 dose-dependently blocked the TGF-β1 activated conversion of CD8^+^ T cells into CD8^+^ Tregs. These data suggested that *in vitro*-induction of CD8^+^ Tregs depended in part on TGF-β1 activation of p38 MAPK signaling. Therefore, p38 MAPK could be a therapeutic target in OC anti-tumor immunotherapy.

## INTRODUCTION

Ovarian cancer (OC) is a significant cause of cancer-related mortality among gynecological malignancies. Approximately 75%-80% of OC patients are in stage III/IV disease at time of diagnosis because early disease is largely asymptomatic [1]. Patients with advanced OC exhibit a progressively deficient immune response which is believed to be associated with its poor outcome. Recent studies have suggested that tumors capable of creating an immunosuppressive microenvironment can escape immune surveillance and undergo progression [2, 3]. Therefore, a better understanding of how an immunosuppressive microenvironment develops and functions in cancer is required.

Regulatory T cells (Tregs) are potent immuno-suppressive cells. Tregs are highly enriched in the tumor microenvironment and are pivotal mediators of immune suppression [4]. We and others have previously demonstrated that Treg populations are elevated in patients with OC and other tumors [5–7]. Furthermore, the presence of tumor-infiltrating Tregs correlates with poor prognosis in many tumor types [8–10]. Therefore, Tregs represent a potential target for disruption of local immune suppression in tumor immunotherapy [11].

CD4^+^ Tregs and CD8^+^ Tregs are the two main Treg cell subtypes. Compared with CD4^+^ Tregs, little is known about the role of CD8^+^ Tregs in tumor immunity. Recent studies indicate that CD8^+^ Tregs are associated with cancer progression and immune evasion [12–14]. The transcription factor forkhead box protein P3 (Foxp3) is a specific intracellular marker of Tregs and is crucial for appropriate Treg differentiation and function [15]. CD25 is the interleukin (IL)-2 receptor α-chain and also a phenotypic marker of CD8^+^ Tregs. Yukiko et al. reported that prostate tumor-derived CD8^+^ Tregs express both CD25 and Foxp3 [16]. We have further revealed that human OC SKOV3 cells could convert CD8^+^ effector T cells into suppressive CD8^+^ Tregs *in vitro* [5]. These findings indicated that CD8^+^ Tregs could be induced by the tumor microenvironment. However, the mechanisms involved are poorly understood.

The immune-modulating cytokine transforming growth factor beta 1 (TGF-β1) is a critical factor in the regulation of both cell differentiation and induction of immune tolerance [17]. TGF-β1 can also induce Tregs [18, 19]. TGF-β1 promotes the expansion of Treg populations by converting conventional CD4^+^CD25^−^ T cells into Foxp3-expressing iTregs [20–22]. Furthermore, the TGF-β signaling pathway is implicated in the promotion of Treg immunosuppressive function [23]. Here, we provide the first evidence that OC cells induce CD8^+^ Tregs by secreting TGF-β1.

We examined changes to CD8^+^ Treg phenotypic marker expression and tumor suppressive mechanisms in response to changes in TGF-β1 levels and the tumor microenvironment. We found that neutralization of TGF-β1 partially counteracted the phenotypic and immunosuppressive function changes of CD8^+^ Tregs normally observed in the co-culture system of CD8+ cells and OC cells. Levels of CD8^+^ Tregs and TGF-β1 were also measured in OC patients to further demonstrate correlation between the two markers. We investigated a critical role of altered TGF-β1 signaling in CD8^+^ Tregs. This study increased our understanding of TGF-β1 as a therapeutic target for cancer therapy.

## RESULTS

### Neutralization of TGF-β1 partially counteracts induction of CD8^+^ Tregs in OC microenvironment

**Figure 1:**
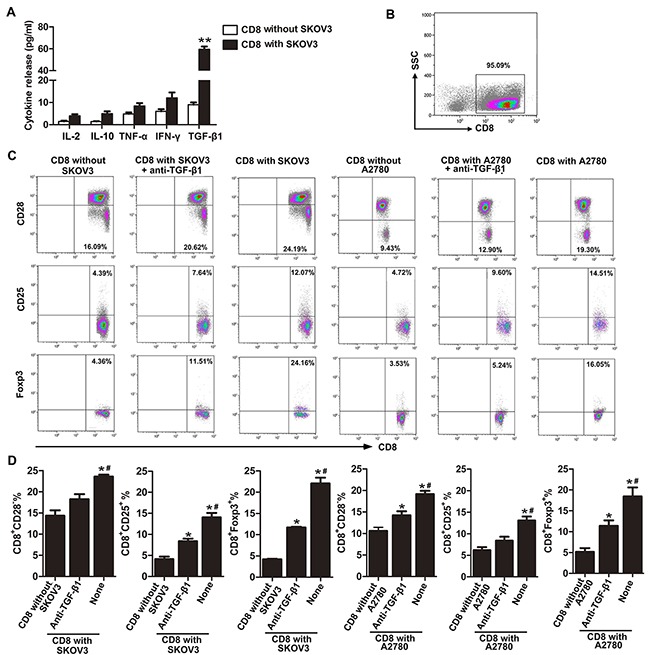
Neutralization of TGF-β1 partially counteracts induction of CD8^+^ Tregs in OC microenvironment **A.** Production of cytokines in medium of co-culture system that CD8^+^ T cells cultured with/without SKOV3 were assessed by ELISA.**P< 0.01. **B.** Isolated cell purity was determined by flow cytometry using PE-conjugated anti-CD8 antibody. **C.** Representative flow cytometry analysis of CD28, CD25, and Foxp3 staining of CD8^+^ T cells co-cultured with SKOV3/A2780 cells alone or in the presence of anti-TGF-β1 neutralizing antibody. Control CD8^+^ T cells are also indicated. **D.** Percentages of CD8^+^CD28^−^, CD8^+^CD25^+^, and CD8^+^Foxp3^+^ T cells in SKOV3/A2780 coculture, SKOV3/A2780 coculture with anti-TGF-β1 neutralizing Ab and CD8^+^ T cells cultured alone group. *P<0.05 compared with CD8^+^ T cells cultured alone. ^#^*P*<0.05 compared with SKOV3/A2780 co-cultured with TGF-β1-neutralizing antibody.

We have previously shown that CD8^+^ Tregs can be induced from peripheral blood CD8^+^ T cells by co-culture with OC cell lines. Here, we further explore the possible mechanism of CD8^+^ Treg induction in the tumor microenvironment using an *in vitro* transwell culturing system. Our results showed that TGF-β1 levels in co-cultured supernatant of CD8^+^ T cells with SKOV3 were significantly increased than in CD8^+^ T cells alone (Figure 1A). Meanwhile, levels of IL-2, IL-10, TNF-α, and INF-γ were not significantly different between the two groups.

We then investigated whether the TGF-β1 in CD8^+^ T cell and SKOV3 co-cultured supernatant was required for induction of CD8^+^ Tregs by using a TGF-β1-neutralizing antibody. Neutralization of TGF-β1 markedly reduced the percentages of CD8^+^CD28^−^ Tregs, CD8^+^CD25^+^ Tregs, and CD8^+^Foxp3^+^ Tregs when compared with CD8^+^ T cells cultured with SKOV3 cells (Figures 1C and 1D). However, our results also showed that this neutralizing effect was not complete because the percentages of CD8^+^CD25^+^ Tregs, and CD8^+^Foxp3^+^ Tregs in TGF-β1-neutralizing group were still higher than that in the control. Similar results were observed using the OC cell line A2780 (Figures 1C and 1D). These results revealed that TGF-β1 may serve as an important activator of CD8^+^ Treg induction in the SKOV3/A2780 co-culture system.

### Blockade of TGF-β1 abrogates the suppressive function of CD8^+^ T cells in the co-culture system

**Figure 2:**
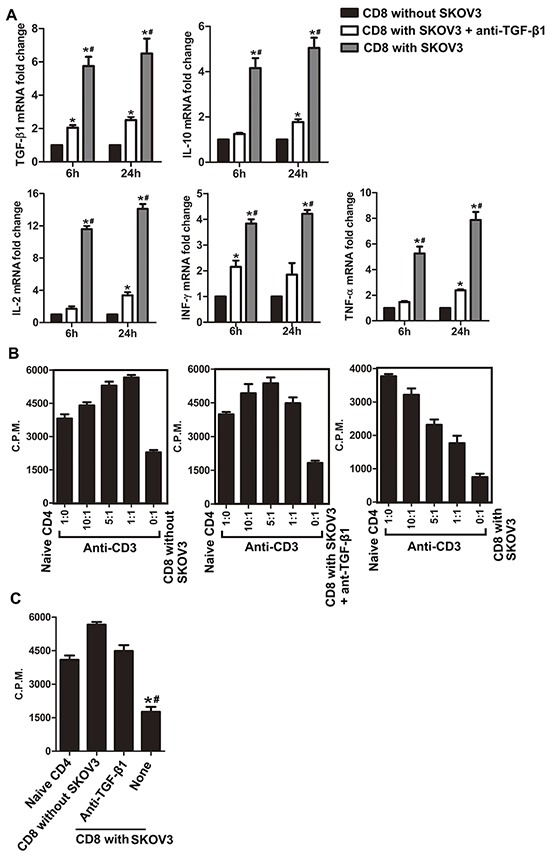
Blockade of TGF-β1 abrogates the suppressive function of CD8^+^ T cells in the co-culture system **A.** TGF-β1, IL-10, IFN-γ, IL-2, and TNF-α mRNA expression levels in CD8^+^ T cells isolated from the co-culture system after co-stimulation with anti-CD3 and anti-CD28 antibodies for 6 and 24 h. *P<0.05 compared with CD8^+^ T cells cultured alone. ^#^*P*<0.05 compared with SKOV3 cells co-cultured with anti-TGF-β1 neutralizing antibody. **B.** CD8^+^ T cells cultured with SKOV3 suppressed naïve CD4^+^ T cell proliferation in a dose-dependent manner with stimulation by anti-CD3 mAb. Conversely, CD8^+^ T cells co-cultured with SKOV3 in the presence of TGF-β1-neutralizing antibody or CD8^+^ T cells cultured alone did not have the suppressive activity. The proliferative response was determined by [^3^H] thymidine uptake. **C.** Suppressive effects of cocultured CD8^+^ T cells on naïve CD4^+^ T cells was analyzed in a ratio of 1:1, the suppressive effect of CD8^+^ T cells cultured with SKOV3 was in part blocked when the assay was performed in the presence of TGF-β1-neutralizing antibody. Results are expressed as mean cpm ± SD of three independent experiments. *P<0.05 compared with CD8^+^ T cells cultured alone. ^#^*P*<0.05 compared with SKOV3 cells co-cultured with TGF-β1-neutralizing antibody.

In addition to examining the capacity of TGF-β1 to change the phenotypes of CD8^+^ T cells in the co-culture system with SKOV3/A2780, we further studied the effect of TGF-β1 on the expression of immunosuppressive cytokines by CD8^+^ T cells in the co-culture system. Neutralization of TGF-β1 markedly decreased production of IL-2, IL-10, TNF-α, INF-γ, and TGF-β1 in CD8^+^ T cells cultured with SKOV3 (Figure 2A). However, compared with CD8^+^ T cells cultured alone, levels of these suppressive cytokines in TGF-β1-neutralizing group were still increased at different time points.

We next explored the ability of TGF-β1 to influence the suppressor activity of Tregs *in vitro*. Naïve CD4^+^ T cells were markedly suppressed by CD8^+^ T cells co-cultured with SKOV3 cells in a dose-dependent manner (Figure 2B, right panels). Contrastingly, no suppressive activity was exhibited by CD8^+^ T cells co-cultured with SKOV3 in the presence of TGF-β1-neutralizing antibody (Figure 2B, middle panels) or CD8^+^ T cells alone (Figure 2B, left panels). Furthermore, co-culture of CD8^+^ T cells and naïve CD4^+^ T cells at a ratio of 1:1 revealed that the suppressive function of CD8^+^ T cells co-cultured with SKOV3 cells could be partially blocked by TGF-β1-neutralizing antibody (Figure 2C). Similar results were obtained using A2780 cells (Supplementary Figure 2), which revealed that TGF-β1 may play a more important role in suppressive function of CD8^+^ Treg cells induced by SKOV3/A2780. Together with data presented in Figure 1, these results indicated that TGF-β1 may be at least partially responsible for the differentiation and suppressive functions of CD8^+^ Tregs induced in OC microenvironment.

### TGF-β1 highly expressed in OC tissues

**Figure 3:**
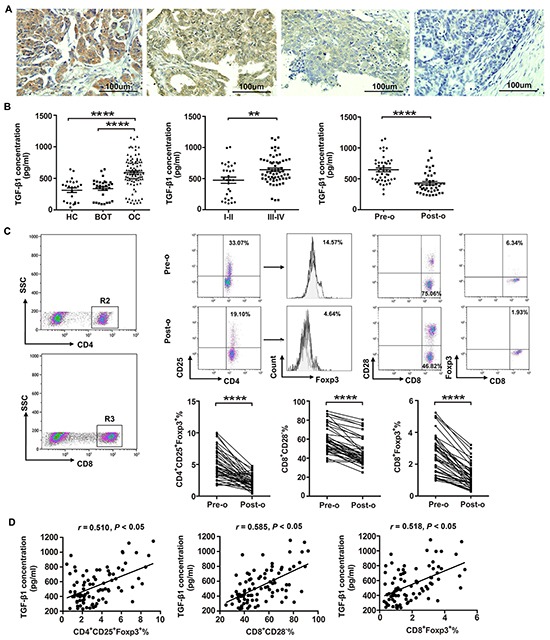
High levels of Tregs and TGF-β1 are present in patients with ovarian cancer **A.** Representative images depicting immunohistochemical staining of TGF-β1 in ovarian cancer tumor tissues. Original magnification: × 200. **B.** Levels of TGF-β1 in patients with OC (n=92), BOT (n=31), and healthy controls (HC; n=24) were determined by ELISA. ****P<0.0001 compared with BOT or HC groups. Cumulative data showed that TGF-β1 levels in OC patients at stage III/IV (n=63) was higher than that in patients at stage I/II (n=29). **P<0.01. **C.** CD8^+^ and CD4^+^ T cells were selected for further analysis by flow cytometry. Representative dot plot of CD4^+^CD25^+^Foxp3^+^, CD8^+^CD28^−^, and CD8^+^Foxp3^+^ T cell levels in peripheral blood from patient pre-operation (pre-o) and post-operation (post-o). ****P<0.0001. Statistical analysis showed that the percentages of CD4^+^CD25^+^Foxp3^+^, CD8^+^CD28^−^, and CD8^+^Foxp3^+^ Treg cells are significantly dropped in OC patients after surgery. ****P<0.0001. **D.** Pearson correlation analysis between the percentages of CD4^+^CD25^+^Foxp3^+^, CD8^+^CD28^−^, and CD8^+^Foxp3^+^ Treg cells and TGF-β1 expression level in OC patients. *P<0.05.

Having shown that OC cells’ induction effect on CD8^+^ Treg cells was activated by TGF-β1, we asked whether TGF-β1 highly expressed in OC tissues. As shown in Figure 3A, TGF-β1 was mainly present in the cell membrane and cytoplasm. The expression rate of TGF-β1 in OC tissues (82.9%, 34/41) was significantly higher than that in OBT (35%, 7/20) and BOT (20%, 4/20) tissues. However, there was no significant difference in TGF-β1 expression between borderline and benign groups (Table 1). We also evaluated the relationships between TGF-β1 expression and clinicopathological factors such as pathological type, FIGO stage, tumor differentiation, and lymphatic metastasis. Table 2 showed that TGF-β1 expression was positively correlated with the FIGO stage (*p*=0.022) and lymphatic metastasis (*p*=0.01).

**Table 1:** The expression of TGF-β1 in different ovarian tissues

Group	n	TGF-β1				Positive (%)	*P* value
		-	+	++	+++		
Ovarian cancer	41	7	6	19	9	82.9	
Ovarian borderline tumor	20	13	3	4	0	35	< 0.001^Δ^
Benign ovarian tumor	20	16	4	0	0	20	< 0.001^*^

Δ Ovarian cancer vs. Ovarian borderline tumor

* Ovarian cancer vs. Benign ovarian tumor

**Table 2:** Correlations of TGF-β1 expression with clinicopathological characteristics of ovarian cancer

Features	TGF-β1			
	n	Positive cases (n)	Rate(%)	*P*
Pathological type				
Mucous	15	13	86.6	0.629
Serous	26	21	80.77	
FIGO stage				
I-II	14	9	64.3	0.022
III-IV	27	25	92.6	
Differentiation grade				
High	19	17	89.5	0.301
Middle/Low	22	17	77.3	
Lymphatic matastasis				
No	23	16	69	0.01
Yes	18	18	100	

### Level of TGF-β1 in OC patients correlated with percentage of CD8^+^ Tregs

We used ELISA to detect plasma levels of TGF-β1 in 92 OC patients, 31 age-matched BOT patients, and 24 healthy controls (Table 3). TGF-β1 levels were significantly higher in OC patients compared with both BOT patients and healthy controls (Figure 3B; *P*<0.0001). Furthermore, patients with stage III/IV OC had significantly elevated TGF-β1 (*P*<0.01) compared with those patients with stage I/II (Figure 3B).

**Table 3:** The clinicopathologic characteristics of the ovarian patients, benign ovarian tumor patients and healthy controls

	Patients		
	OC	BOT	HC
Number	92	31	24
Age ± SD^a^(y)	57.41 ± 19.21	46.87 ± 17.13	49.24 ± 19.76
*Stage* (n)^b^			
I/II	29		
III/IV	63		
*Histology (n)*			
Serous	41		
Mucinous	32		
Others^c^	19		

Abbreviations: OC, ovarian cancer; BOT, benign ovarian tumor; HC, healthy controls.

a Age at diagnosis.

b According to the International Federation of Gynecology and Obstetrics.

c Including poorly differentiated adenocarcinoma and mesonephroid carcinoma.

We further assessed the TGF-β1 levels and the percentage of CD8^+^ Tregs in the 44 OC patients before and after surgery using ELISA and flow cytometry. Levels of TGF-β1 dropped significantly following surgery (Figure 3B, *P*<0.0001). Additionally, we observed that percentages of CD4^+^CD25^+^Foxp3^+^, CD8^+^CD28^−^ and CD8^+^Foxp3^+^ Tregs were significantly lower following surgery (Figure 3C, *P*<0.0001). Pearson correlation analysis showed that CD4^+^CD25^+^Foxp3^+^, CD8^+^CD28^−^ and CD8^+^Foxp3^+^ Treg proportions were positively correlated with TGF-β1 levels (*r* = 0.510, *P*<0.05; *r* = 0.585, *P*<0.05; *r* = 0.518, *P*<0.05, respectively; Figure 3D).

### P38 MAPK signaling is required for the conversion of CD8^+^ T cells to CD8^+^ Tregs in OC microenvironment

**Figure 4:**
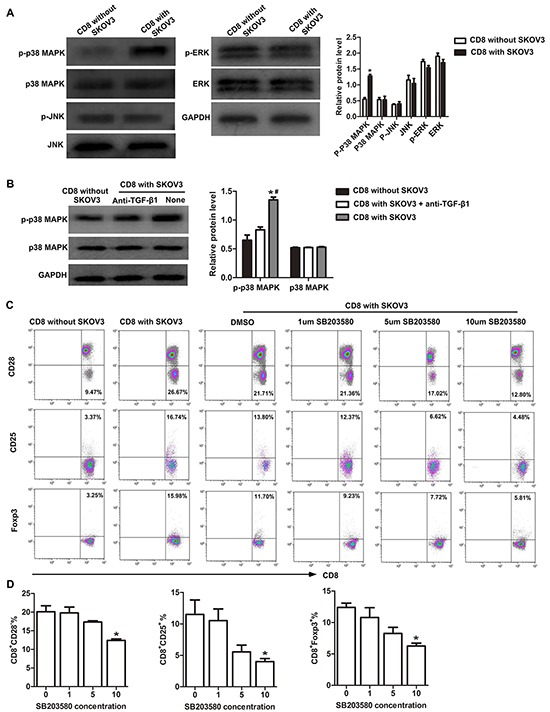
p38 MAPK signaling is required for conversion of CD8^+^ T cells to CD8^+^ Tregs **A.** Western blotting analysis of the expression levels of p-p38 MAPK, p38 MAPK, p-ERK, ERK, p-JNK, and JNK in CD8^+^ T cells co-cultured with or without SKOV3 cells. *P<0.05. **B.** Western blotting analysis of the expression levels of p-p38 MAPK and p38 MAPK in CD8^+^ T cells co-cultured with SKOV3 cells in the presence or absence of TGF-β1-neutralizing antibody. *P<0.05 compared with CD8^+^ T cells cultured alone. ^#^*P*<0.05 compared with SKOV3 cells with TGF-β1-neutralizing antibody. **C.** Representative flow cytometry analysis of CD28, CD25, and Foxp3 staining of CD8^+^T cells co-cultured with SKOV3 cells and treated with different concentrations of SB203580. **D.** Inhibition of p38 was performed by adding different concentrations of SB203580 to the primary co-culture system, SB203580 dramatically inhibited the conversion of CD8^+^ Tregs induced by TGF-β1 in a dose-dependent manner. *P<0.05.

Next, we determined the signaling pathways involved in TGF-β1-activated induction of CD8^+^ Tregs. Expression of phosphorylation of p38 MAPK (p-p38MAPK) increased significantly (*P*<0.05) in CD8^+^ T cells cultured with SKOV3 cells compared with in CD8^+^ T cells alone. Expression of p38 MAPK appeared similar in both groups. There was also no significant differences in expression of JNK or ERK phosphorylation between these two groups (Figure 4A). In TGF-β1-neutralizing experiment, we further found that phosphorylation of p38 MAPK in CD8^+^ T cells cultured with SKOV3 cells was significantly inhibited by TGF-β1-neutralizing antibody (Figure 4B). Moreover, the p38-specific inhibitor SB203580 impaired TGF-β1-activated CD8^+^ Treg differentiation (Figure 4C) and dramatically inhibited the conversion of CD8^+^ Tregs induced by TGF-β1 in a dose-dependent manner (Figure 4D). These results suggested that TGF-β1 secreted by OC cells may induce CD8^+^ Tregs through the p38 MAPK signaling pathway.

## DISCUSSION

Several mechanisms of tumor-mediated immunesuppression have been identified, including Treg infiltration, expression of B7-H1 and B7-H4 coinhibitory molecules by tumor cells and tumor-associated antigen-presenting cells [26]. Here, we focus on the role of CD8^+^ Treg in tumor immunity. Tumor cells could directly induce CD8^+^ Treg population expansion, and these tumor-induced Tregs were associated with reduced survival and increased mortality [27]. Therefore, understanding the mechanisms underlying Treg induction is critical for the development of effective biologic therapies. TGF-β1 is a potent immunosuppressive cytokine. Dandawate *et al*. reported that TGF-β1 played a significant role in the induction and expansion of Tregs in both tumors and the periphery [28, 29].

We previously reported that CD8^+^ Tregs could be induced from CD8^+^ T cells by co-culture with human OC cells SKOV3/A2780 cells [5]. However, the mechanism responsible was not identified. In this study, we found increased secretion of TGF-β1 by SKOV3 *in vitro*. Meanwhile, little or no IL-2, IL-10, TNF-α, or INF-γ was detected. Therefore, we assessed whether the increased TGF-β1 in the OC microenvironment was important for CD8^+^ Treg induction from CD8^+^ T cells. Chen *et al.* reported that TGF-β1 secreted by OC cells could generate CD4^+^CD25^+^ Treg cells with hyporesponsive and suppressive features [30]. Additionally, Eusebio *et al*. detected an increased frequency of CD8^+^CD25^+^Foxp3^bright^ Tregs in asthma patients when TGF-β1 levels were increased [31]. We demonstrated that TGF-β1-neutralizing antibody significantly decreased percentages of CD8^+^CD28^−^, CD8^+^CD25^+^ and CD8^+^Foxp3^+^ Tregs. Importantly, TGF-β1-neutralizing antibody also inhibited the immunosuppressive function of *in vitro-*induced CD8^+^ Tregs.

We also found TGF-β1 highly expressed in OC tissues and plasma of OC patients. The level of TGF-β1 also correlated with tumor stage and lymph node metastasis in OC patients. Interestingly, we observed a positive correlation between TGF-β1 and Tregs in OC patients, with both the the level of TGF-β1 and percentages of Tregs decreased significantly following surgery. The present results indirectly suggested the relationship of TGF-β1 and Tregs in OC patients and TGF-β1 maybe involved in OC progression.

TGF-β1 can activate downstream signaling mechanisms including the three major MAPK pathways, p38, ERK and JNK in several cell types [32]. Currently, the role of MAPK signaling in TGF-β1-activated iTreg is controversial. One study has shown that ERK played a central role in the generation of the Treg phenotype in naïve T cells following TGF-β1 stimulation, while p38 was not necessary [33]. However, Huber *et al*. reported that p38 MAPK signaling was required for the induction of TGF-β1-activated iTregs [34]. Additionally, Adler *et al*. demonstrated that the regulatory function of iTregs was associated with an enhanced p38 MAPK activity [35]. Consistently, we found that p-p38 MAPK activity was upregulated in CD8^+^ T cells cultured with SKOV3, while ERK and JNK were not significantly differences. Furthermore, activation of p-p38 in CD8^+^ Tregs was significantly inhibited by addition of TGF-β1-neutralizing antibody, suggesting that p38 MAPK signaling is functionally important for the differentiation of TGF-β1-activated CD8^+^ Tregs. We therefore analyzed Treg markers of CD8^+^ T cells cultured in the presence of a p38 MAPK inhibitor SB203580, which suppressed CD8^+^ Treg differentiation in a dose-dependent manner. These findings supported a role for p38 MAPK signaling in the differentiation of TGF-β1-activated CD8^+^ Tregs.

Higher levels of TGF-β1 are associated with an increased proportion of Tregs in OC patients. TGF-β1 secreted by OC cells stimulates CD8^+^ T cells to acquire a regulatory function and phenotype. Activation of p38 MAPK signaling may increase the induction of TGF-β1-activated CD8^+^ Tregs. Our findings revealed a novel mechanism of immune tolerance induced by OC cells, suggesting that specific inhibition of Tregs could lead to better therapeutic strategies of ovarian cancer.

## MATERIALS AND METHODS

### Patients and specimens

This study was approved by the Ethical Committee of the First Affiliated Hospital of Nanjing Medical University (Nanjing, China). Written informed consent was obtained from each participant. Pre-operative blood samples from patients who received no prior treatment were collected from 92 new cases with OC and 31 new cases with benign ovarian tumor (BOT) at the First Affiliated Hospital of Nanjing Medical University. Forty-four patients in the OC group underwent surgery and post-operation blood samples were collected after 1 month. Twenty-four age-matched healthy donors with no family history of autoimmune disease or tumors served as controls. For immunohistochemistry assays, OC tissues (n=41), ovarian borderline tumor (OBT; n=20) and BOT (n=20) samples were obtained from the pathology department of Nanjing Maternity and Child Health Care Hospital from May 2011 to August 2015. All tissue sections were diagnosed by specialists. Tumor tissues were staged according to the tumor-node-metastasis system of the International Federation of Gynecology and Obstetrics. Patient characteristics are displayed in Table 1.

### Blood sample collection and CD8^+^ T cell isolation

Venous blood was collected from OC and BOT patients and healthy donors using EDTA tubes. Plasma was isolated and stored at −70°C prior to measuring level of TGF-β1. Peripheral blood mononuclear cells (PBMCs) were separated by Ficoll-Hypaque density gradient centrifugation (GE Health Care Life Sciences, Piscataway, NJ, USA). Next, CD8^+^ T cells were isolated from PBMCs using a CD8-positive isolation kit (Dynal, Oslo, Norway). Isolated cell purity was > 95% (Figure 1B) as determined by flow cytometry using PE-conjugated anti-CD8 antibody (Beckman, Marseille, France).

### Cell culture and culture conditions

The human OC cell lines SKOV3 and A2780 were purchased from the American Type Culture Collection (Manassas, VA, USA) and KeyGEN (Nanjing, China), respectively. SKOV3 and A2780 cells were grown in McCoy's 5A (Invitrogen, Carlsbad, CA) or RPMI 1640 (Gibco, Gaithersburg, MD) medium supplemented with 10% fetal bovine serum (FBS; Invitrogen, Carlsbad, CA, USA) in a 5% CO_2_ at 37°C.

### Co-culture of CD8^+^ T cells with SKOV3/A2780 cells

Transwell co-culture experiments were performed in 24-well plates with 0.4 μm pore sizes in inner wells (Greiner, Frickenhausen, Germany) to physically separate CD8^+^ T cells and SKOV3/A2780 cells. Approximately 2 × 10^5^ SKOV3/A2780 cells per well were grown in the outer wells using 1.5 mL 1640-medium containing 10% human AB serum. Next, 6 × 10^5^ isolated human CD8^+^ T cells were added into the inner wells in 500 μL of the same medium. For TGF-β1 neutralization experiments, 10 μg/mL anti-TGF-β1 neutralizing antibody (R&D Systems, Minneapolis, MN, USA) was added to the SKOV3/A2780 co-culture system to block TGF-β1 function. After 5 days incubation, CD8^+^ T cells were harvested and washed with PBS. Approximately 1 × 10^6^ cells were collected for flow cytometry analysis, and 2 × 10^6^ cells were collected for western blot analysis. The remaining cells were dispensed into 96-well plates at 4×10^4^ cells per well and stimulated with 1 mg/mL soluble anti-CD3 and anti-CD28 antibodies (eBioscience, San Diego, CA, USA). After 6 and 24 h stimulation, CD8^+^ T cells were collected for evaluation of cytokine mRNA expression levels by RT-PCR.

### Flow cytometry analysis

*In vitro* cultured cells or PBMCs from patient blood samples were washed and stained with a combination of fluorochrome-conjugated monoclonal antibodies. PE-anti-CD4, PE-anti-CD8, APC-anti-CD25, and APC-anti-CD28 antibodies (all from BD Biosciences, San Jose, CA, USA) were used. Labeled cells were incubated away from light for 20 min at room temperature. Next, cells were washed and stained with FITC-conjugated anti-Foxp3 (eBioscience) after fixation and permeabilization according to the manufacturer's instructions. All events were acquired using a Gallios flow cytometer (Beckman Coulter, Brea, CA, USA) and data were analyzed using Kaluza 1.3 software (Beckman Coulter).

### Enzyme-linked immunosorbent assay (ELISA) and cytometric bead array (CBA)

TGF-β1 levels in culture supernatants and plasma were determined using an ELISA Kit (eBioscience). IL-2, IL-10, tumor necrosis factor (TNF)-α, and interferon (IFN)-γ levels in culture supernatants were quantified using the Human Th1/Th2 Cytokine CBA kit (BD Biosciences). All protocols were conducted according to the manufacturer's instructions

### RNA isolation and quantitative real-time PCR

Total RNA was isolated from CD8^+^ T cells using the miRNeasy Mini Kit (Qiagen, Germantown, MD, USA). PrimeScript RT Master Mix (Takara, Otsu, Japan) was used for reverse transcription following the manufacturer's standard protocol. Cytokine expression levels were determined using an ABI 7500 system (Life Technologies, Foster City, CA, USA) and SYBR Green. Primer sequences used were TGF-β1: 5′-CAGCAACAATTCCTGGCGATAC-3′ and 5′-TCAACCACTGCCGCACAACT-3′; IFN-γ: 5′-GTTT TGGGTTCTCTTGGCTGTTA-3′ and 5′-AAAAGAGTTC CATTATCCGCTACATC-3′; TNF-α: 5′-CCCCAGGGACC TCTCTCTAATC-3′ and 5′-GGTTTGCTACAACATGG GCTACA-3′; IL-2: 5′-GAATGGAATTAATAATTACA AGAATCCC-3′ and 5′-TGTTTCAGATCCCTT TAGTT CCAG-3′; IL-10: 5′-GCTGGAGGACTTTAAGGGTTA CCT-3′ and 5′-CTTGATGTCTGGGTCTTGGTTCT-3′; β-actin: 5′-GAGCTACGAGCTGCCTGACG-3′ and 5′-GT AGTTTCGTGGATGCCACAG-3′. Cycle threshold (CT) values were estimated by normalizing these values against β-action CT values using the 2^−^^ΔΔCt^ method.

### Proliferation assay for naïve CD4^+^ T cells

CD8^+^ T cells cultured under desired conditions were harvested. The Dynabeads Untouched Human CD4 T Cells and Dynabeads FlowComp Human CD45RA Kits (Dynal), enabled isolation of naïve CD4^+^ T cells from healthy donors. CD8^+^ T cells collected from the three groups were co-cultured with naïve CD4^+^ T cells at ratios of 1:0, 1:1, 1:5, 1:10, and 0:1 in 96-well plates for 56 h. Anti-CD3 antibody (500 ng/mL;eBioscience) and irradiated PBMCs were added to the culture in a final volume of 200 μL/well. After 16 h of pulsing with 2.5 μCi/mL [^3^H] thymidine, cells were harvested and the proliferation of naïve CD4^+^ T cells were analyzed by scintillation counting.

### Immunohistochemistry

Sections to be stained were incubated with 3% H_2_O_2_ for 15 min to inhibit endogenous peroxidase activity, followed by 2 h blocking with normal goat serum at room temperature. Anti-TGF-β1 antibody (1:100) was incubated with the blocked sections for 18 h at 4°C. Next, HRP-labeled goat-antimouse IgG antibody (1:2000) was applied for 30 min at 37°C. Finally, these actions were colored with diaminobenzidine tetrahydrochloride solution and then counter-stained with Mayer'shematoxylin. Slides were mounted and observed with an optical microscope (BX51; Olympus, Tokyo, Japan). Negative controls were processed in an identical manner, omitting the primary antibodies. The scoring system for immunostained sections is based both on staining intensity and the percentage of immunoreactive cells, and is described elsewhere [24, 25]. Briefly, staining intensity was scored 0–3 (0, negative; 1, weak; 2, moderate; 3, strong). The percentage of immunoreactive cells documented as 0 (less than 5%), 1 (6–25%), 2 (26–50%), 3 (51–75%), and 4 (76–100%). For each section, the two resultant numbers were multiplied and a score attained. A score of 0–2 was defined as negative (−), 3 or 4 as weakly positive (+), 5–8 as moderately positive (++), and 9–12 as strongly positive (+++).

### Western blot analysis

Protein from co-cultured cells was extracted using the protease inhibitor cocktail and phosphatase inhibitor cocktail (Kangchen Bio-tech, China). Cell lysates were subjected to SDS-PAGE electrophoresis and proteins were blotted onto PVDF membranes. Appropriate primary antibodies and corresponding HRP-conjugated secondary antibodies were used. Exposure was performed using enhanced chemiluminescence reagents (Millipore, Billerica, MA, USA) and x-ray film. Antibodies used in western blotting were: anti-p38, anti-phospho-Thr180/Tyr182 p38, anti-ERK1/2, anti-phospho-Thr202/Tyr204 ERK1/2, anti-JNK, anti-phospho-Thr183/Tyr185 SAPK/JNK, and anti-GAPDH (all from Cell Signaling Technology).

### Inhibition of p38 mitogen-activated protein kinase (MAPK)

Inhibition of p38 was performed by adding different concentrations of SB203580 (Cell Signaling Technology) to the primary co-culture system described above. DMSO was added to control cultures at equivalent concentrations. CD8^+^ T cells were collected at day 5 for analysis of Foxp3, CD25, and CD28 levels by flow cytometry.

### Statistical analysis

All data were analyzed using SPSS 16.0 software and the means ± SD of at least three independent experiments were presented. One-way analysis of variance was performed for multiple comparisons. An independent samples *t*-test was used for analyzing the differences between two groups. Changes to Treg and TGF-β1 levels in patients who underwent surgery were assessed by paired-sample *t*-test. Pearson correlation analysis was performed to evaluate correlations. A *P* value < 0.05 was considered to be statistically significant.

## SUPPLEMENTARY FIGURES


