# Supplementary material for: TGF-β1 contributes to CD8+ Treg induction through p38 MAPK signaling in ovarian cancer microenvironment
Source: Oncotarget. 2016 Jun 14;7(28):44534–44. doi: 10.18632/oncotarget.10003 (PMC5190116; doi:10.18632/oncotarget.10003)
Supplement: Supplementary file 1 [file oncotarget-07-44534-s001.pdf]

TGF-β1 contributes to CD8⁺ Treg induction through p38 MAPK signaling in ovarian cancer microenvironment

SUPPLEMENTARY FIGURES

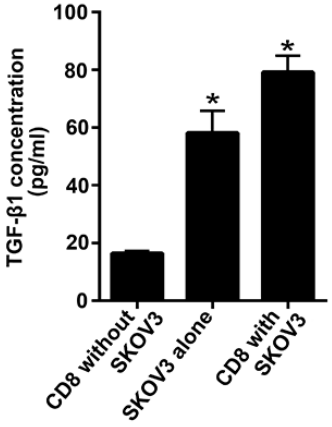

Supplementary Figure S1: Expression of TGF-β1 in CD8⁺ T cells and SKOV3 co-culture supernatants. Production of TGF-β1 in co-culture supernatants that SKOV3 cultured alone and CD8⁺ T cells cultured with/without SKOV3 were detected by ELISA, *P<0.05.

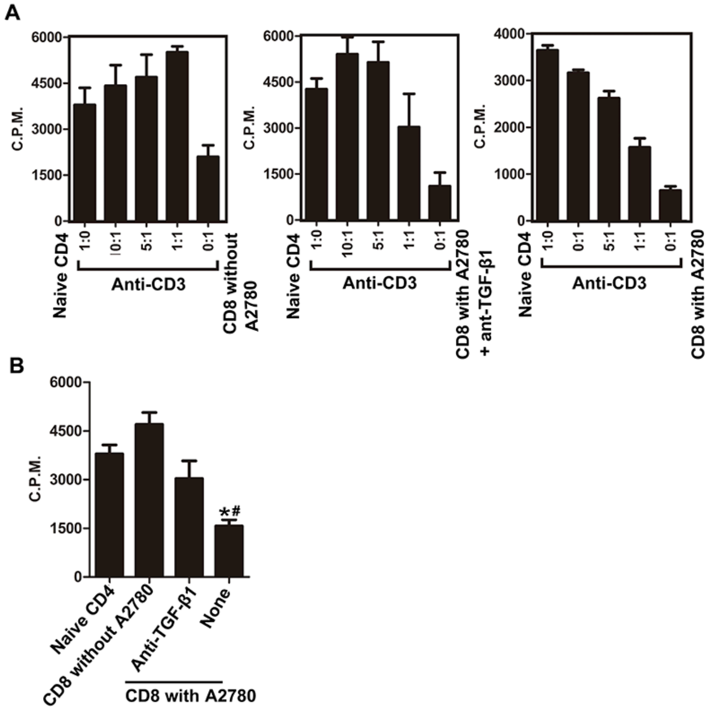

Supplementary Figure S2: Blockade of TGF-β1 abrogates the suppressive function of CD8⁺ T cells from the co-culture system A. CD8⁺ T cells cultured with A2780 suppressed naïve CD4⁺ T cell proliferation in a dose-dependent manner with stimulation by anti-CD3 mAb. Conversely, CD8⁺ T cells co-cultured with A2780 in the presence of TGF-β1-neutralizing antibody or CD8⁺ T cells cultured alone did not have the suppressive activity. The proliferative response was determined by [^{3}H] thymidine uptake. **B.** Suppressive effects of cocultured CD8⁺ T cells on naïve CD4⁺ T cells was analyzed in a ratio of 1:1, the suppressor effect of CD8⁺ T cells cultured with A2780 was in part blocked when the assay was performed in the presence of TGF-β1-neutralizing antibody. Results are expressed as mean cpm ± SEM of three independent experiments. *P<0.05 compared with CD8⁺ T cells cultured alone. $^{\#}P$<0.05 compared with A2780 cells co-cultured with TGF-β1-neutralizing antibody.

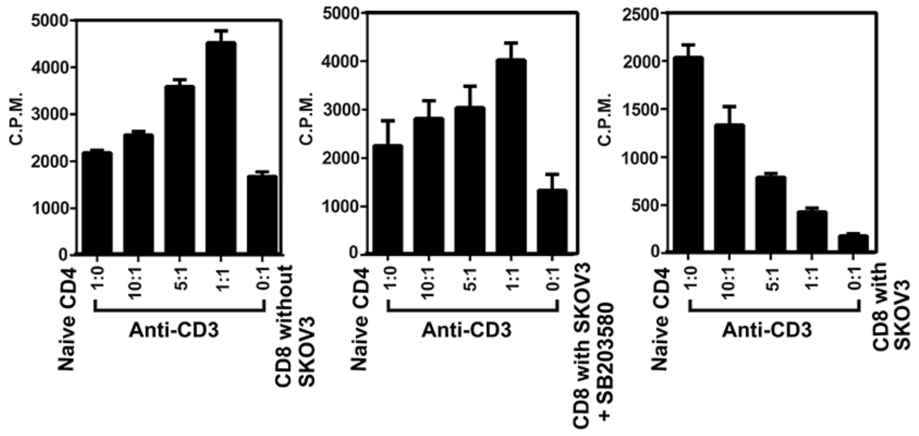

Supplementary Figure S3: p38 MAPK inhibitor SB203580 inhibit the suppressive function of CD8$^+$ Tregs on naïve CD4$^+$ T cells. CD8$^+$ T cells were treated with 10 μM p38-specific inhibitor SB203580 for 2 h, and then cultured with OC cell line SKOV3. CD8$^+$ T cells co-cultured with SKOV3 possess significant suppressive activity for naïve CD4$^+$ T cells. No suppressive activity was exhibited by SB203580 treated CD8$^+$ T cells or CD8$^+$ T cells alone.

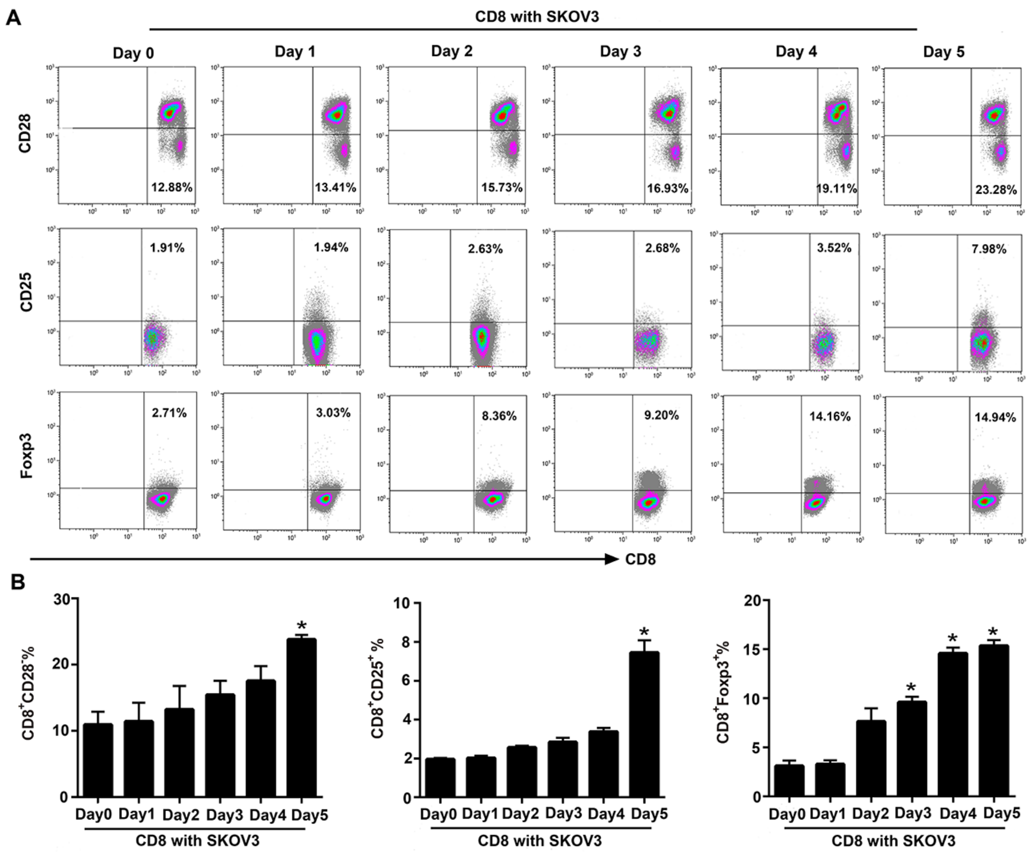

Supplementary Figure S4: CD8$^+$FoxP3$^+$Tregs is more easily induced by tumor-derived TGF-β1. A. Representative flow cytometry analysis of CD28, CD25, and Foxp3 staining of CD8$^+$T cells co-cultured with SKOV3 cells in five consecutive days. **B.** The percentages of CD8$^+$FoxP3$^+$Tregs was significant increased in day 3 in CD8$^+$ T cell cultured with SKOV3 system compared with CD8$^+$ T cell in day 0, while the percentages of CD8$^+$CD28$^-$ Tregs and CD8$^+$CD25$^+$ Tregs were significant increased in day 5.
